# Gene expression study in monocytes: evidence of inflammatory dysregulation in early-onset obsessive-compulsive disorder

**DOI:** 10.1038/s41398-022-01905-1

**Published:** 2022-03-31

**Authors:** Natalia Rodríguez, Luisa Lázaro, Ana E. Ortiz, Astrid Morer, Albert Martínez-Pinteño, Alex G. Segura, Patricia Gassó, Sergi Mas

**Affiliations:** 1 Department of Basic Clinical Practice, University of Barcelona, Barcelona, Spain; 2 Department of Child and Adolescent Psychiatry and Psychology, Institute of Neurosciences, Hospital Clinic de Barcelona, Barcelona, Spain; 3 Department of Medicine, University of Barcelona, Barcelona, Spain; 4 Institut d’Investigacions Biomèdiques August Pi i Sunyer (IDIBAPS), Barcelona, Spain; 5 Centro de Investigación Biomédica en Red de Salud Mental (CIBERSAM), Barcelona, Spain

**Keywords:** Psychiatric disorders, Comparative genomics, Pathogenesis

## Abstract

Obsessive-compulsive disorder (OCD) has a complex etiology that seems to include immune dysfunction and alterations in circulating monocytes. To investigate the immune basis and the functional dysregulation of monocytes in this disease, we analyzed gene expression in the peripheral monocytes of pediatric patients with OCD (*N* = 102) compared to controls (*N* = 47). We examined gene expression in primary cultures of peripheral monocytes from participants, under basal conditions and under exposure to lipopolysaccharide (LPS) to stimulate immune response. Whole-genome expression was assessed in 8 patients and 8 controls. Differentially expressed genes were identified followed by protein-protein interaction network construction and functional annotation analysis to identify the genes and biological processes that are altered in the monocytes of OCD patients. We also explored the expression levels of selected genes in monocytes from the other participants using qPCR. Several changes in gene expression were observed in the monocytes of OCD patients, with several immune processes involved under basal conditions (antigen processing and presentation, regulation of immune system and leukocyte cell adhesion) and after LPS stimulation (immune and inflammatory response, cytokine production and leukocyte activation). Despite the qPCR analysis provided no significant differences between patients and controls, high correlations were observed between the expression levels of some of the genes and inflammatory markers (i.e., T helper 17 and regulatory T cell levels, total monocyte and proinflammatory monocyte subset levels, and the cytokine production by resting and stimulated monocytes) of the study participants. Our findings provide more evidence of the involvement of monocyte dysregulation in early-onset OCD, indicating a proinflammatory predisposition and an enhanced immune response to environmental triggers.

## Introduction

Obsessive-compulsive disorder (OCD) is a common neuropsychiatric disease with a lifetime prevalence of 1–3% [1]. It is a heterogeneous condition characterized by recurrent and persistent thoughts (obsessions), and repetitive and ritualistic behaviors (compulsions), developing in childhood (early onset) in approximately 30–50% of patients [2, 3]. Childhood-onset OCD may represent a distinct neurodevelopmental disorder in which different etiopathogenic mechanisms are involved [4].

Although the pathophysiology of OCD remains poorly understood, multiple studies support the role of a significant genetic contribution as well as environmental factors, with an estimated heritability at about 30% [5]. A growing body of evidence suggests that this gene-environment interaction could lead to a dysfunction of the immune system (involving both innate and adaptative immunity arms) in some individuals, which would contribute to the etiopathogenesis of the disease [6–8]. Several studies have identified some susceptibility genes for OCD related to the immune system [9–12]. Further evidence is seen with the incidence of concurrent autoimmune comorbidities and triggering infectious events [6, 13], presence of anti-neuronal antibodies and inflammatory processes within the cortico-striatal circuits [14–16], imbalance in lymphocyte subpopulations [17], and altered proinflammatory cytokine levels [18–21] in OCD patients. In some individuals, these abnormalities emerge early in children, suggesting a role of immune dysregulation in early-onset OCD.

In a recent study by our group, we observed increased levels of circulating monocytes and a higher percentage of proinflammatory monocyte subsets in children with OCD. Purified monocytes from these patients also produced a larger amount of inflammatory cytokines upon immune stimulation with lipopolysaccharide (LPS) [22]. Blood monocytes are among the main cells of the innate immune system and take on important roles in phagocytosis, cytokine production, and T cell activation via antigen presentation [23]. Over recent decades, attention has been devoted to the possible contribution of these cells in the development and progression of neuropsychiatric disorders because monocytes and the effectors they secrete can not only drive inflammation and cause tissue damage but also influence synaptic transmission, neural plasticity, and brain function under both physiological and pathological states [24, 25].

Gene expression constitutes an intermediate measure between genetic susceptibility and clinical phenotype and can be considered an indicator of cellular function. Most genes act in a coordinated manner with other genes to influence a particular phenotype, thereby defining a network of gene interactions. Thus, gene expression profiling and gene network analysis allow the identification of both differentially expressed genes and the dysregulated molecular pathways involved in the etiopathogenesis of disease [26]. In the present study, we aimed to investigate the functional alteration of monocytes in pediatric patients with OCD by focusing on the analysis of gene expression profiles in this cell type both under basal conditions and after an immune stimulation with LPS.

## Material and methods

### Study design

**Fig. 1:**
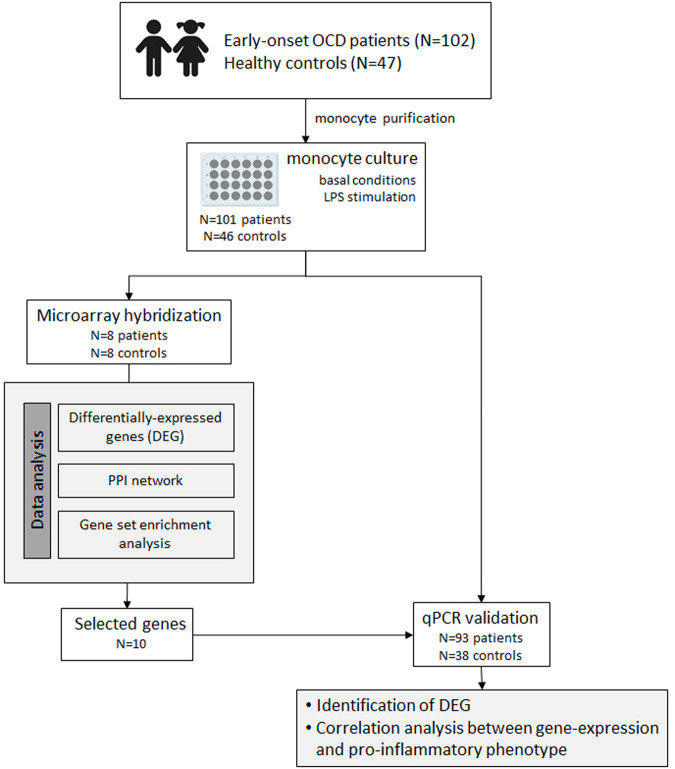
Overview of the study. DEG differentially expressed genes, LPS lipopolysaccharide, OCD obsessive-compulsive disorder, PPI protein-protein interaction.

We conducted a whole-genome analysis of differences in gene expression from microarray hybridization in a subset of patients. The analytical methodology was based on protein-protein interaction network construction and functional annotation analysis to explore the genes and biological processes altered in monocytes from OCD patients. To validate our findings, we then assessed the expression levels of selected genes in monocytes from the remaining individuals in our cohort of patients with OCD and controls using qPCR to assess the differences in gene expression between both groups and to explore the correlations between these gene expression levels and the proinflammatory phenotype of these individuals. An overview of the study design is shown in Fig. 1.

### Subjects

The sample comprised 102 patients with OCD and 47 healthy controls of both genders, ranging in age from 8 to 19, and recruited from the Department of Child and Adolescent Psychiatry and Psychology at the Hospital Clínic in Barcelona between 2010 and 2014 [22, 27]. All cases were diagnosed with OCD according to the criteria of the Diagnostic and Statistical Manual of Mental Disorders, fourth edition (DSM-IV) [28]. The Spanish version [29] of the Schedule for Affective Disorders and Schizophrenia for School-age Children-Present and Lifetime Version (K-SADS-PL) interview [30] was used to confirm the diagnosis of OCD and any additional current or past psychiatric diseases. This interview was administered with both parent(s) and the child as informants. Due to the naturalistic study design, patients could receive antidepressants and/or cognitive-behavioral therapy based on the clinical guidelines of the hospital.

The healthy control group was recruited from schools in the same geographical region. Controls and their parents were interviewed with the Spanish version [29] of the K-SADS-PL to assess current and past psychopathology. We excluded potential cases and controls if they suffered intellectual disability, neurological disorders, or known inflammatory disease. Children with a personal history of psychiatric disorders were also excluded from the control group.

Demographic and clinical data of the study population are shown in Table 1. All participants denied alcohol and other recreational drug use during the semi-structured interviews. All procedures were approved by the hospital’s Ethics Committee. Written informed consent was obtained from all parents and verbal informed consent was given by all subjects following an explanation of the procedures involved.

**Table 1:** Demographic and clinical data of the study population.

	OCD patients (*N* = 102)	Controls (*N* = 47)	Statistic, *p* value
Gender (female/male) N (%)	50 (49.0)/52 (51.0)	33 (70.2)/14 (29.8)	χ^2^ = 5.86, *p* = 0.016
Age (mean ± SEM)	14.78 ± 0.26	16.29 ± 0.26	*t* = −3.608, *p* = 0.0004
Age of onset (mean ± SEM)	12.95 ± 0.28	–	–
Duration of illness, months (mean ± SEM)	24.62 ± 2.44	–	–
CY-BOCS score (mean ± SEM)	25.97 ± 0.64	–	–
Comorbidities, N (%)			
Anxiety or mood disorders	46 (45.1)	–	–
ADHD or tic disorder	22 (21.6)	–	–
Treatment, N (%)			
Medicated	82 (80.4)	–	–
Antidepressants	59 (72)	–	–
Antipsychotics	1 (1.2)	–	–
Antidepressants + antipsychotics	22 (26.8)	–	–
Non-medicated	20 (19.6)	–	–

*OCD* obsessive-compulsive disorder, *ADHD* attention deficit hyperactivity disorder, *CY-BOCS* Children’s Yale-Brown Obsessive-Compulsive Scale, *SEM* standard error of the mean.

### Sample collection and culture of purified monocytes

Blood samples from all OCD patients and controls were collected using BD Vacutainer tubes containing Acid Citrate Dextrose (Becton Dickinson, Franklin Lakes, New Jersey, USA). Peripheral blood mononuclear cell (PBMC) suspensions were prepared by density gradient centrifugation over Ficoll-Plaque (GE Healthcare Bio-Science AB, Uppsala, Sweden) at 750 × *g* for 20 min at 18 °C. After washing, cells were frozen in fetal bovine serum (FBS) (Life Technologies, Carlsbad, CA, USA) containing 10% dimethyl sulfoxide (Sigma-Aldrich, St. Louis, MO, USA) and stored in liquid nitrogen until subsequent analysis.

On the day of testing, monocytes were purified from PBMCs and cultured as described previously [22]. Briefly, after thawing PBMCs, monocytes were purified by negative selection using an indirect magnetic labeling system (MACS, Miltenyi Biotec, Auburn, CA, USA) following the manufacturer’s protocol. Then, the purified monocytes were seeded at a density of 1.5 × 10^5^ cells/well on 24-well plates and allowed to rest for 2 h in RPMI 1640 medium supplemented with 2 mM L-glutamine, 10% FBS, 100 units/mL penicillin and 100 µg/mL streptomycin (Life Technologies, Carlsbad, CA, USA) before immune stimulation with 1 ng/mL LPS (Sigma-Aldrich, Saint Louis, MO, USA). LPS was dissolved in culture medium, which was used as a vehicle for the negative control. After 24 h of incubation in a humidified incubator with 5% CO_2_ at 37 °C, cells were washed and rapidly frozen at −80 °C. No monocytes were purified from one sample from each group (a patient with OCD and a healthy control) despite following the same protocol. Hence, we used 101 samples from patients with OCD and 46 samples from healthy controls for the primary culture of monocytes.

### RNA extraction and quality control

After cell thawing, monocyte pellets were homogenized in TRIzol reagent (Life Technologies, Foster City, CA, USA) and RNA was isolated following the manufacturer’s instructions. RNA quantity and quality were determined using a Nanodrop ND-2000 spectrophotometer (NanoDrop, Wilmington, DE, USA). An Agilent 2100 Bioanalyzer (Agilent Technologies, Palo Alto, CA, USA) was used to assess the integrity of RNA. RNA samples were prepared for the gene expression analysis as described below.

### Gene expression analysis by microarray

#### Microarray hybridization

Eight samples from patients with OCD and eight from healthy controls, under both basal conditions and after LPS stimulation, were used for genome-wide expression analysis using microarray. Samples were selected based on our previous results of immune dysregulation in monocytes from our cohort of OCD patients and controls [22], using the monocyte samples from patients who had higher inflammatory values (i.e., percentage of total monocytes and proinflammatory CD16^+^ monocytes, and their cytokine production levels in primary cultures) and control individuals with lower inflammatory properties. A description of the inflammatory characteristics of the selected samples is shown in Supplementary Table S1.

RNA samples were prepared for microarray hybridization using GeneChip Pico Reagent Kit (Affymetrix, Santa Clara, CA, USA). After labeling and amplification, the cDNA was hybridized to the Affymetrix Human PrimeView Array Plate (Affymetrix, Santa Clara, CA, USA), according to the manufacturer’s protocol. Sample processing and hybridization was performed at the Kompetenzzentrum für Fluoreszente Bioanalytik (KFB, BioPark Regensburg GmbH, Regensburg, Germany). Two samples (one case and one control, both after LPS stimulation) were not hybridized due to cDNA quality and amount. A total of 30 samples could be hybridized in the array, including 16 samples from untreated monocytes (8 patients and 8 controls) and 14 from LPS-stimulated monocytes (7 patients and 7 controls).

#### Microarray data analysis

Full details of the extraction, labeling and hybridization protocols as well as the raw array data (.cel files) and the pre-processed data matrix will be available at the Gene Expression Omnibus database (http://www.ncbi.nlm.nih.gov/geo/).

Microarray analysis was performed using the Babelomics 5 suite (http://www.babelomics.org/) [31]. The data were normalized using robust multichip analysis. Multiple probes mapping to the same gene were merged using the average as the summary of the hybridization values. Differential gene expression was detected using the Limma package from Babelomics. To account for multiple testing effects, *p*-values were corrected using the false discovery rate (FDR). The gene expression fold change (FC) was also calculated. Genes with a corrected *p*-value < 0.01 and an absolute log_2_FC ≥ 2 were considered significantly differentially expressed.

#### Protein-protein interaction (PPI) network construction and evaluation

The top differentially expressed genes between cases and controls (FDR-corrected *p*-value < 0.01, absolute log_2_FC ≥ 2) before and after LPS stimulation were used to create PPI networks with the Search Tool for the Retrieval of Interacting Genes/Proteins (STRING) database (www.string-db.org-). This tool integrates a large number of known and predicted protein interactions [32]. Only curated information and data derived from experimental evidence were used to construct the networks. The obtained PPI networks were visualized and analyzed using Cytoscape 3.5.1 [33]. For each node, the degree of connectivity (i.e., the number of edges or direct interactions a particular node has) and the betweenness centrality, which is related to the existence of hubs connecting different parts of the network, were computed.

#### Gene set enrichment analysis and visualization

To provide a functional interpretation of the different transcriptional activities identified between the monocytes from patients and controls, a gene set enrichment analysis was performed using the Database for Annotation, Visualization and Integrated Discovery (DAVID) (http://david.ncifcrf.gov/) [34]. The functional annotation tool in DAVID provides the most relevant biological processes enriched within a given gene list, using Gene Ontology (GO) terms [35], and groups similar annotation terms in significantly enriched clusters of functional categories to reduce redundancy. Functional clusters with an enrichment score > 2 (the geometric mean, in -log scale, of the member’s *p*-values in a corresponding annotation cluster) and containing ≥ 3 significant GO terms (FDR-corrected *p*-value < 0.05) were considered statistically significant. The significantly enriched clusters were used to construct a functional network, generated using the FGNet package in R-Bioconductor, to visualize the associations and overlap between clusters [36].

### Gene expression analysis by Fluidigm Biomark System

Among the differentially expressed genes identified between cases and controls, under both basal conditions and after LPS stimulation, 10 genes were selected from each condition (Supplementary Table S2) for further analysis of gene expression in the remaining RNA samples (93 OCD patients and 38 controls). For the priorization of the genes, a functional approach was followed, considering not only the magnitude of the gene expression changes but also the functional role of the genes. Hence, gene selection was based on the following: (a) the *p*-value obtained from the microarray data analysis (the top 10% of the genes with the minimum *p*-value received one point); (b) the FC obtained from the microarray data analysis (the top 10% of the genes with the highest absolute FC received one point); (c) their relevance in PPI networks (hub genes according to the degree of connectivity received one point and hub genes according to betweenness centrality received one point); (d) their functional relevance (genes included in the top five significantly enriched functional clusters received one point); and (e) their expression in microglial cells (genes expressed in microglia received one point). Scores could range from 0 to 6, and genes with the highest scores were selected for further analysis (all with a score ≥4).

For the gene expression analysis, 50 ng of purified RNA were reverse transcribed into cDNA using Fluidigm® Reverse Transcription Master Mix (Fluidigm Inc., Palo Alto, CA, USA) and amplified in a target-specific amplification using PreAmp Master Mix (Fluidigm Corporation, South San Francisco, CA, USA) and TaqMan® Gene Expression Assays (Applied Biosystems, Foster City, CA, USA) for 16 cycles. The qPCR reactions were then performed in the Biomark HD instrument using Flex Six IFC (Fluidigm) and TaqMan® Gene Expression Assays (Applied Biosystems, Foster City, CA, USA). *PPIA*, a commonly used housekeeping gene with a stable gene expression in the microarray data, was included as an endogenous control. Real-Time PCR Analysis software 4.5.2 (Fluidigm) was used to visualize results and perform quality control with a quality threshold set to 0.60, baseline correction set to linear (derivative) and Ct threshold method set to auto (global). Genes and samples that did not meet the validation criteria in more than one-third of the assays were removed from the analysis. This resulted in seven genes under basal conditions and nine after LPS stimulation, with 80–100 samples per gene. Relative expression of the target genes was determined using the ΔΔCt method.

### Statistical analysis

Sample size and statistical power calculation were performed following the recommendations of Wei et al. [37]. Given the sample size of the microarray data analysis, it was possible to identify with more than 80% statistical power changes in gene expression with an absolute log_2_FC ≥ 2 and a two-sided significance level *p* < 0.0001 in the 75% least variable genes. For the qPCR analysis, the sample size ensured a statistical power >80% to detect gene expression changes with a FC ≥ 2 and a two-sided significance level *p* < 0.05 in the 75% least variable genes.

Data were analyzed using IBM SPSS statistics version 20 (IBM Corp., Armonk, NY, USA). Kolmogorov-Smirnov and Shapiro-Wilk tests were used to assess normality, and the equality of variance between groups was assessed using the Levene’s test. Variables following a non-normal distribution were natural log-transformed for subsequent analysis. Differences between the demographic characteristics of patients and controls were assessed by Chi-square or Student *t* tests, according to the distribution and scales of the variables. To identify clinical variables that might affect gene expression, Pearson’s correlation test, Student *t* test or ANOVA were used, as appropriate. Pearson correlation was also used to explore the correlation between relative expression of the target genes analyzed with Fluidigm and the immune parameters previously reported to be altered in our sample of patients with OCD (i.e., percentage of total monocytes, percentage of proinflammatory CD16^+^ monocytes, cytokine production in primary monocyte cultures, percentage of T helper 17 (Th17) cells, and percentage of regulatory T cells (Treg)) [17, 22]. The significance level was set at *p* < 0.05 (two-tailed).

## Results

### Differential gene expression in microarrays

The gene expression analysis of the monocyte samples under basal conditions showed significant differences in the expression levels of 258 genes between patients with OCD and healthy controls. Besides, 139 differentially expressed genes were found when monocytes from patients and controls were analyzed after LPS stimulation. The list of differentially expressed genes in basal and stimulated conditions, with their corresponding FC and FDR-corrected *p*-values, is shown in Supplementary Table S3. Seventy-five of these genes were overlapped between both conditions (29% and 54%, respectively).

### PPI network construction

**Fig. 2:**
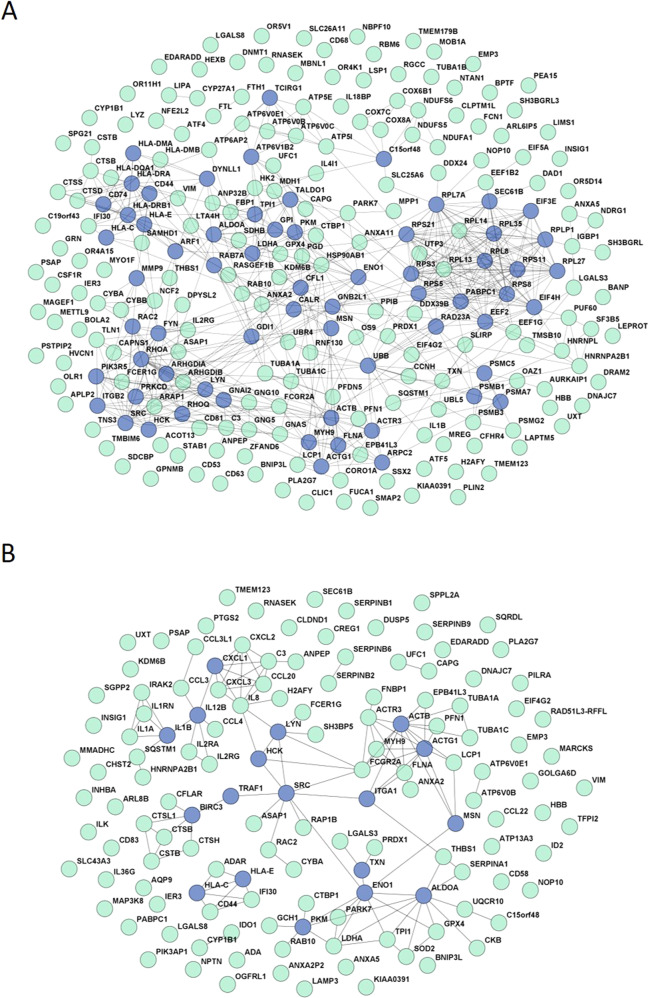
Protein-protein interaction network. Protein‑protein interaction network of differentially expressed genes in untreated monocytes (**A**) and after stimulating monocytes with LPS (**B**) The spheres represent the genes (nodes) and the lines represent the direct interactions (edges) between differentially expressed genes. Hub genes are shown in dark blue.

The top differentially expressed genes (FDR-corrected *p*-value < 0.01 and absolute log_2_FC ≥ 2) between cases and controls under basal conditions and after LPS stimulation were used to construct the PPI networks. The untreated monocyte network comprised 252 genes from the initial list (97.6%) and 616 edges with 176 interacting genes (Fig. 2A). As shown in Fig. 2B, the interaction network for the LPS-stimulated monocytes included 138 nodes from the initial list (99.3%) and 132 edges with 83 interacting genes. The networks obtained with each dataset showed significant PPI enrichment *p*-values (basal conditions *p* = 1 × 10^−16^; LPS stimulation *p* = 4.4 × 10^−16^), indicating significantly more interactions between the nodes than expected for a random gene set of similar size in the genome.

### Gene set enrichment analysis

**Fig. 3:**
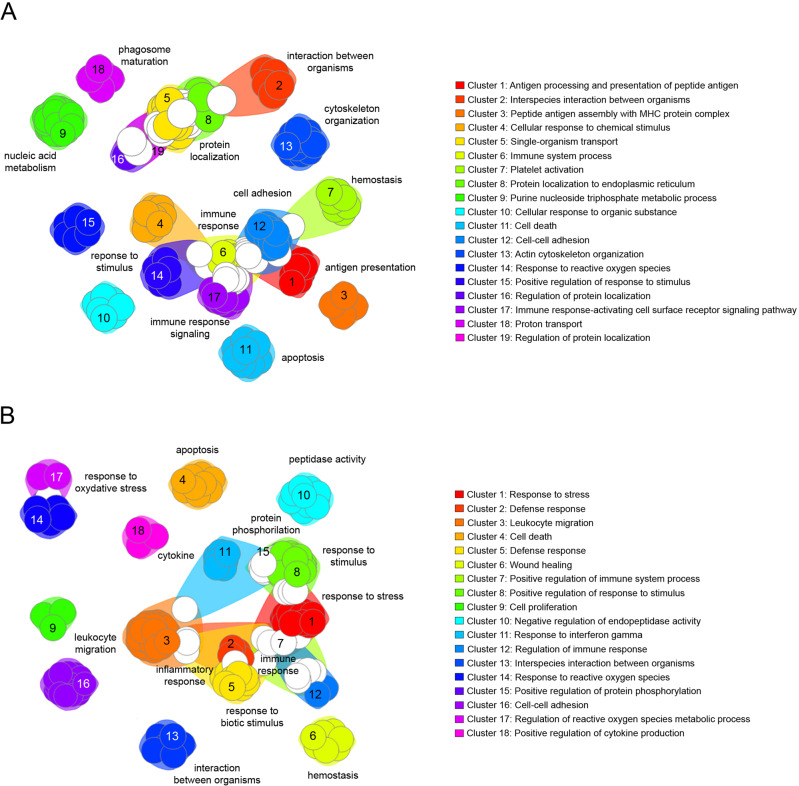
Gene set enrichment analysis. Functional network with the significantly enriched clusters of Gene Ontology biological processes (GO) according to DAVID for the genes differentially expressed in untreated monocytes (**A**) and after LPS stimulation (**B**) The network was constructed using FGNet package in R. Each node corresponds to a specific GO term; terms within the same cluster are placed together surrounded by a common background color. Terms that appear in only one functional cluster are plotted with the color of the cluster background whereas terms that are included in more than one cluster are left in white. The clusters are numbered by order of significant enrichment. The most significant biological process is indicated for each cluster.

To provide a functional interpretation of the genes differentially expressed between cases and controls under both basal conditions and after LPS stimulation, clusters of functionally related terms (GO terms) enriched in each gene list were explored using DAVID. The functional analysis identified 19 and 18 significantly enriched clusters when analyzing the gene list from unstimulated and LPS-stimulated monocytes, respectively (Supplementary Table S4). Several immune processes were included under each condition. In untreated monocytes, the top functional categories were related to antigen processing and peptide antigen presentation, which were the main biological processes in clusters 1 and 3. The other most significant clusters included GO terms for response to chemical and organic stimuli (clusters 4, 10, 14, and 15), regulation of the immune system and immune response (clusters 6 and 17), and leukocyte cell adhesion (cluster 12). The remaining terms were related to more specific functions, such as apoptosis or hemostasis, or more general processes related to protein transport and localization, actin cytoskeleton organization, and nucleic acid metabolism (Fig. 3A). Among LPS-stimulated monocytes, the functional analysis of the differentially expressed genes showed that most of the genes were related to the host defense response, including immunity, and the innate immune response in particular (clusters 1, 2, 7, and 12); inflammatory response (clusters 2, 3, and 5); response to stress (clusters 1, 2, and 14); responses to chemical, organic, and biotic stimuli (clusters 1 and 5); response to cytokines (clusters 1, 11, and 18); and leukocyte migration and proliferation (clusters 3, 9, 11, and 16), among others. As observed under basal conditions, other terms related to apoptosis, hemostasis, peptidase activity, or regulation of protein phosphorylation were also included in the top clusters (Fig. 3B).

### Gene expression analysis by Fluidigm

**Fig. 4:**
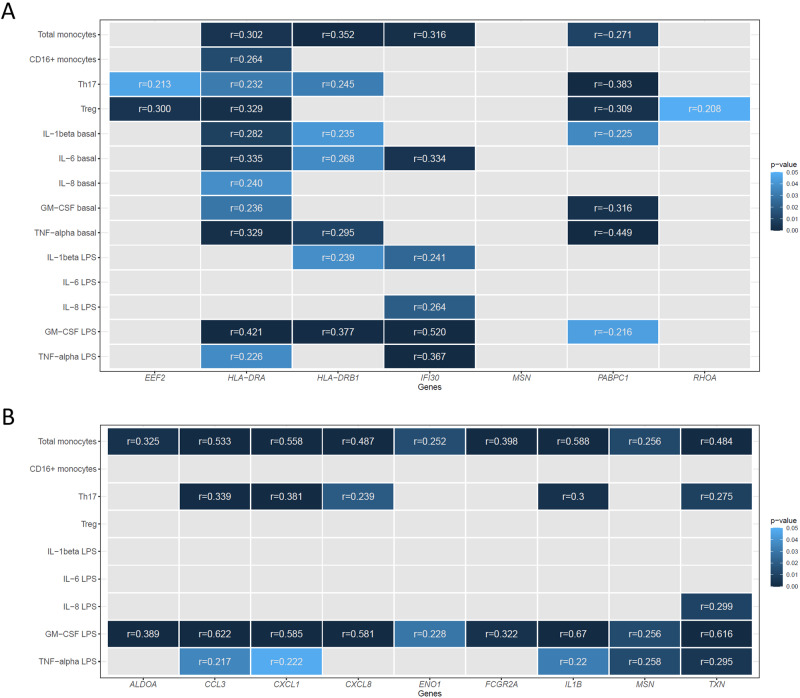
Correlations between gene expression and immune parameters. The heatmap shows the correlation between the expression levels of the genes assessed in basal conditions (**A**) and after LPS stimulation (**B**) with the immune parameters that were previously reported to be altered in our cohort of OCD patients (percentage of total monocytes and proinflammatory CD16 + monocytes, percentage of T helper 17 (Th17) and regulatory T cells (Treg), and cytokine production in primary cultures of monocytes under both conditions). Pearson correlations were computed for pairs of continuous variables. Only significant correlations (*p* < 0.05) are shown.

We further assessed the gene expression of ten selected genes differentially expressed in each condition (unstimulated and LPS-stimulated monocytes) in the remaining cases and controls. The expression levels of the different genes assessed in each condition were significantly correlated (*p* < 0.05) (Supplementary Fig. S1). None of the demographic and clinical factors assessed that could have an impact on gene expression, such as age, gender, disease severity, use of psychoactive medications or presence of psychiatric comorbidities, showed a significant association with the expression levels of the different genes explored. No significant differences were found either in the gene expression levels between patients and controls (results not shown). However, a significant correlation was observed between the gene expression of the monocyte samples in both conditions and some of the immune parameters previously assessed in these individuals (Fig. 4). Specifically, four genes differentially expressed in the microarray data under basal conditions (*HLA-DRA*, *HLA-DRB1*, *IFI30* and *PABPC1*) were highly correlated with different immune measures in the extended cohort, including the levels of Th17 and Treg cells, the levels of circulating monocytes, and the production of proinflammatory cytokines in monocyte cultures before and after LPS-stimulation (*p* < 0.05). In addition, *EEF2* and *RHOA* expression levels correlated significantly with the Th17 and Treg levels (*p* < 0.05). Regarding the LPS-stimulated monocytes, a high correlation was observed between the expression levels of the nine genes analyzed (*ALDOA*, *CCL3*, *CXCL1*, *CXCL8*, *ENO1*, *FCGR2A*, *IL1B*, *MSN* and *TXN*) and the circulating monocyte levels as well as between the gene expression levels and the production of granulocyte-macrophage colony-stimulating factor (GM-CSF) by LPS-stimulated monocytes (*p* < 0.05). In addition, the expression levels of most genes (*CCL3*, *CXCL1*, *CXCL8*, *IL1B*, *MSN*, and *TXN*) were significantly correlated with the levels of Th17 and the production of other proinflammatory cytokines in LPS-stimulated monocytes (*p* < 0.05).

## Discussion

In order to gain a better understanding of the pathophysiology of OCD and the implication of monocyte dysregulation in the development of the disease, we conducted, for the first time to our knowledge, a genome-wide analysis of the differences in gene expression, under basal conditions and after an immune stimulation with LPS, in primary cultures of peripheral monocytes from early-onset OCD patients. We used a systems biology analytical approach based on PPI network construction and functional annotation analysis of the genes expressed differently between cases and controls. In addition, we used extended cohorts of OCD patients and controls to validate the differences in the expression levels of selected genes and to assess the correlation between these gene expression levels and the values of immune parameters explored previously in those individuals. Our findings support the idea that altered monocyte function, based on gene expression profiles, may be involved in the pathophysiology of OCD, at least in a subgroup of patients.

Although no previous work has assessed gene expression profiles in monocytes from patients with OCD, some authors have described functional dysregulation of these immune cells in other psychiatric disorders based on gene expression changes. Thus, monocytes from patients with schizophrenia, bipolar disorder, or major depressive disorder showed abnormal gene expression patterns, with alterations in processes related to immune response, proinflammatory cytokine production, and cell adhesion/migration [38–41]. In addition, genome-wide expression studies using whole blood from those patients found impaired gene expression in some genes mostly related to monocytes and other cells of myeloid lineage [42, 43]. Consistent with these findings, in our study, the microarray analysis in primary cultures of peripheral monocytes from OCD individuals identified a significant alteration in the expression of several genes involved in immune processes and inflammatory responses under basal conditions and after LPS stimulation. The top differentially expressed genes in untreated monocytes were related to antigen processing and presentation, immune system regulation, response to stimuli, and leukocyte cell adhesion. This suggests that monocytes may present some functional dysregulation in OCD patients, even in the absence of external stressors. Previous studies have suggested that genes involved in antigen presentation, such as those from the human leukocyte antigen (HLA) system, may have a role in the etiology of neuropsychiatric diseases [44]. Thus, several variants of HLA genes have been reported as susceptibility factors for psychiatric disorders, such as autism spectrum disorder or schizophrenia [45–48]. Similarly, we recently described a higher frequency of certain *HLA-DRB1* alleles in children with OCD [11]. These genes are involved in antigen presentation to T cells, leukocyte maturation, and immunomodulatory functions, making them essential to the regulation of immune-inflammatory processes [44]. Furthermore, they participate in neurodevelopment and neuroplasticity by regulating microglia and synaptic pruning [49]. Several of these HLA genes (e.g., *HLA-DRB1*, *HLA-DRA*, *HLA-DQA1*, *HLA-DMA*, *HLA-C*, and *HLA-E*), together with other genes involved in antigen presentation and other immune functions, were differentially expressed in the monocytes from OCD patients in our cohort. These basal disturbances in gene expression could lead to an enhanced inflammatory response after exposure to some triggers. In fact, we observed that, upon immune stimulation with LPS, the monocytes of OCD patients exhibited an altered expression of genes involved in processes related to the innate immune response, cytokine production, and leukocyte migration, proliferation, and activation. These results are in accordance with our previous findings, which also showed a greater production of proinflammatory cytokines by monocytes from OCD patients in response to LPS stimulation [22].

We also assessed the expression of selected genes that were differentially expressed in the microarray analysis in the remaining subjects from our cohort. The selection of cases and controls with extreme inflammatory phenotypes for the genome-wide exploration could maximize the differences in gene expression between both groups and, accordingly, milder changes could be expected when analyzing the remaining individuals. Although we were not able to confirm the gene expression differences between patients and controls in this cohort, maybe due to the wide interindividual variability in gene expression levels and the limited sample size, we observed strong correlations between the expression levels of some genes and inflammatory markers found to be significantly altered in OCD patients (i.e., Th17 and Treg cell levels, the levels of total monocytes and proinflammatory monocyte subsets, and monocyte cytokine production before and after LPS stimulation). These results suggest that immune activation of monocytes may not be uniformly increased in OCD individuals, and instead, a gradation of gene expression levels may be present in OCD, affecting especially a subgroup of patients. Thus, we hypothesize that subtle gene expression changes in monocytes from OCD patients could predispose those individuals to present a more pronounced proinflammatory phenotype, which has already been described in these subjects. These gene expression changes may be due in part to the genetic liability to OCD, including variants of different frequency across all chromosomes [5]. Thus, OCD patients with greater changes in gene expression could present a large contribution of rare genetic risk variants, which have a higher impact on pathology by altering gene function.

Hence, our findings suggest that monocytes from OCD patients, at least in a subgroup of individuals, exhibit some degree of proinflammatory predisposition, making them prone to experience an exacerbated inflammatory response upon certain environmental encounters. Given that the immune system is involved in the regulation of brain function, monocyte dysregulation could lead to a series of peripheral and central changes affecting neural circuit development, synaptic plasticity, and neuronal function, thereby facilitating the appearance of behavioral alterations related to OCD [8, 24]. Furthermore, the proinflammatory state of circulating monocytes observed in children with OCD may reflect activation of their brain counterparts, the microglial cells. Indeed, several lines of evidence suggest that microglial dysregulation may have a role in the pathophysiology of OCD and related disorders, such as Tourette syndrome (TS) and autism [50–53]. A recent PET study indicated increased microglial activation in the neurocircuitry of OCD, that correlated with symptom severity [16]. Post-mortem studies evaluating gene expression in basal ganglia from individuals with TS have also identified an upregulation of several microglia-related genes, pointing to microglia proliferation and activation [54, 55]. Interestingly, some genes identified by Lennington et al. were identified among the genes differentially expressed in the peripheral monocytes from our cohort of OCD patients, under both basal conditions (30 genes) and after LPS stimulation (12 genes) [55].

Our findings should be interpreted in the context of several limitations. First, the sample size could limit the statistical power, both in the genome-wide exploration, performed in a subgroup of patients and controls, and in the confirmatory analysis. Second, as previously mentioned, the strategy used to select samples for the microarray analysis could have introduced bias, increasing the identification of gene expression differences. However, this analysis can be considered a first exploration of transcriptomic changes in monocytes from OCD patients. In addition, the further analysis of selected genes by qPCR in the rest of the samples could have been affected by the gene selection approach. Nevertheless, despite the study of the genes with higher gene expression differences could have facilitated the confirmation of the results, the selection strategy used, based on both the gene expression changes and the functional role of the genes, could represent better the monocyte phenotype in OCD patients. Although this exploration did not fully replicate the findings, the correlation between gene expression and proinflammatory profile indicate that monocyte gene expression may be especially altered in a subgroup of patients, with subtle dysregulation of gene expression possibly underpinning the proinflammatory phenotype observed in OCD. Finally, we made no adjustments for multiple comparisons in the analysis of our extended cohort due to the high correlation between the gene expression levels of the different genes and between the inflammatory parameters previously assessed in monocytes and lymphocytes in our cohort. However, even with these limitations, our data provide new evidence of the involvement of monocyte dysregulation in early-onset OCD, being the first study assessing monocyte gene expression in the disease.

We should also stress some strengths of the present study. The gene expression analysis was conceived to explore quantitative and qualitative alterations of gene expression in OCD patients, by microarray analysis using PPI network construction and functional analysis in a subgroup of individuals, followed by replication in an extended cohort. In addition, we used a primary model of purified monocytes instead of whole blood for the gene expression analysis, which allowed us to explore the dysregulation of this cell type in the pathophysiology of OCD specifically and avoid the confounding effects of other immune cells. We should also highlight that our cohort was a well-characterized homogeneous clinical population of children and adolescents with a recent onset of obsessive-compulsive symptoms. This benefits from avoiding the possible influence of important confounding factors such as age, disease duration, or the effect of long-term treatment with antidepressants, and allows the specific etiopathogenic mechanism underlying early-onset OCD to be studied.

In summary, the present study provides new insights into the immune basis of OCD by evidencing the role of monocyte dysregulation in the disease’s pathophysiology. Our results suggest that functional alterations of peripheral monocytes may exist in early-onset OCD, at least in a subgroup of patients, leading to a proinflammatory predisposition and an enhanced immune response to environmental insults. Further exploration is required to replicate these findings. Besides, future studies with larger samples and follow-up information are needed to better characterize the role of monocyte disfunction in the complex pathogenesis of OCD and its contribution to the clinical manifestations of the disease and patient outcomes.

## Supplementary information

Supplementary Figure S1

Supplementary Table S1

Supplementary Table S2

Supplementary Table S3

Supplementary Table S4
