# Supplementary material for: Gene expression study in monocytes: evidence of inflammatory dysregulation in early-onset obsessive-compulsive disorder
Source: Transl Psychiatry. 2022 Mar 31;12:134. doi: 10.1038/s41398-022-01905-1 (PMC8971392; doi:10.1038/s41398-022-01905-1)
Supplement: Supplementary file 1 — Supplementary Figure S1 [file 41398_2022_1905_MOESM1_ESM.pdf]

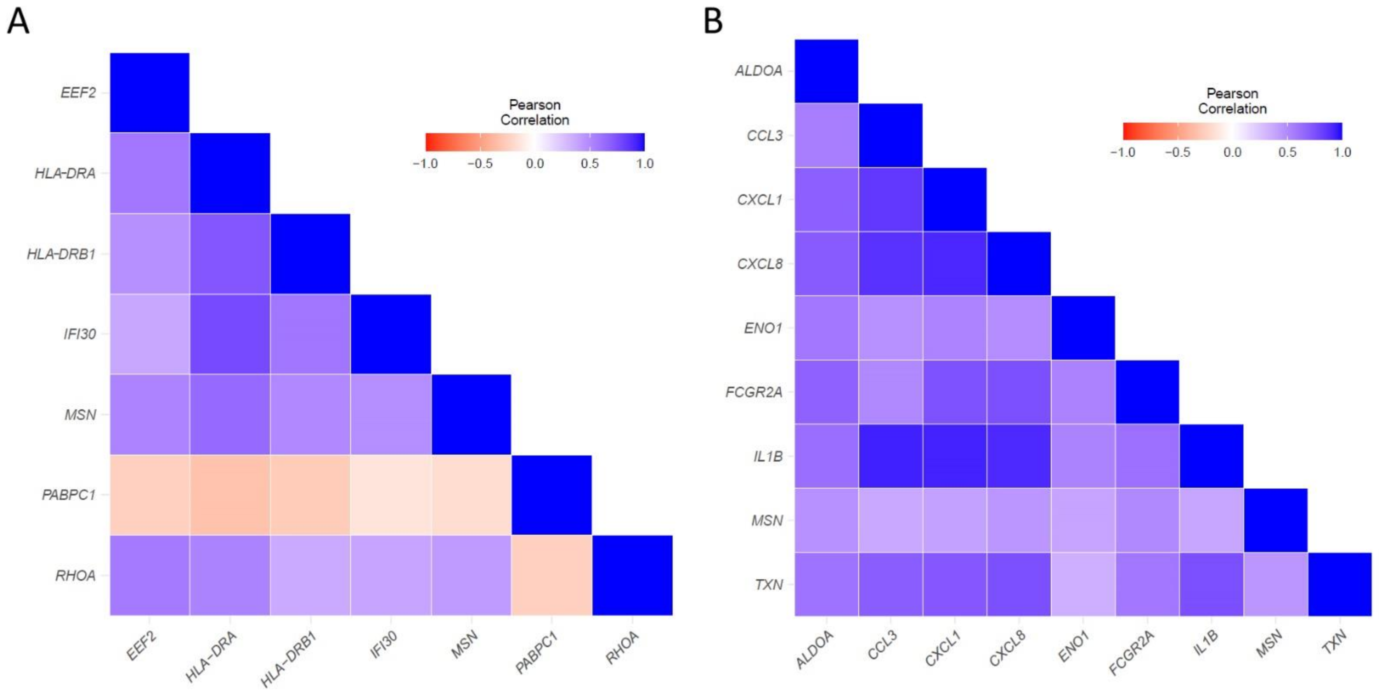

Figure S1. Correlation between expression levels of genes assessed in the extended cohort of OCD patients and controls under both basal conditions (A) and after LPS stimulation (B).
