# Supplementary material for: Gene expression study in monocytes: evidence of inflammatory dysregulation in early-onset obsessive-compulsive disorder
Source: Transl Psychiatry. 2022 Mar 31;12:134. doi: 10.1038/s41398-022-01905-1 (PMC8971392; doi:10.1038/s41398-022-01905-1)
Supplement: Supplementary file 2 — Supplementary Table S1 [file 41398_2022_1905_MOESM2_ESM.pdf]

Table S1. Inflammatory characteristics of the participants included in the microarray analysis.

	OCD patients (N=8)	Controls (N=8)
Total monocytes (%) (mean ± SEM)	5.48 ± 1.32	1.53 ± 0.23
$CD16^+$ monocytes (%) (mean ± SEM)	9.44 ± 0.74	2.98 ± 0.44
IL-1β[a] (mean ± SEM)	1,431.03 ± 174.32	236.99 ± 43.05
IL-6[a] (mean ± SEM)	105,947.08 ± 2,7517.47	7,305.99 ± 3749.84
IL-8[a] (mean ± SEM)	5,507.70 ± 3,101.05	530.95 ± 198.33
GM-CSF[a] (mean ± SEM)	69,921.81 ± 13,591.33	1,974.99 ± 1208.32
TNF-α[a] (mean ± SEM)	69,551.05 ± 11,662.82	1,911.70 ± 723.14

[a] Amount of each cytokine released by total monocytes after stimulation with lipopolysaccharide, expressed as a ratio to that released by non-stimulated cells
