# Supplementary material for: Gene expression study in monocytes: evidence of inflammatory dysregulation in early-onset obsessive-compulsive disorder
Source: Transl Psychiatry. 2022 Mar 31;12:134. doi: 10.1038/s41398-022-01905-1 (PMC8971392; doi:10.1038/s41398-022-01905-1)
Supplement: Supplementary file 3 — Supplementary Table S2 [file 41398_2022_1905_MOESM3_ESM.pdf]

Table S2. Genes selected for further analysis in the extended cohort of OCD patients and controls.

Gene	Gene name	TaqMan Assay ID
**Basal conditions**		
PPIA	peptidylprolyl isomerase A (housekeeping)	Hs99999904_m1
HLA-DMA	major histocompatibility complex, class II, DM alpha	Hs00185435_m1
HLA-DRB1	major histocompatibility complex, class II, DR beta 1	Hs04192464_mH
CD74	CD74 molecule	Hs00269961_m1
PABPC1	poly(A) binding protein cytoplasmic 1	Hs00743792_s1
IFI30	lysosomal thiol reductase	Hs00173838_m1
HLA-DRA	major histocompatibility complex, class II, DR alpha	Hs00219575_m1
MSN	moesin	Hs00792607_mH
RHOA	ras homolog family member A	Hs00357608_m1
ACTG1	actin gamma 1	Hs03044422_g1
EEF2	eukaryotic translation elongation factor 2	Hs00157330_m1
**LPS stimulation**		
PPIA	peptidylprolyl isomerase A (housekeeping)	Hs99999904_m1
IL1B	interleukin 1 beta	Hs01555410_m1
TXN	thioredoxin	Hs00828652_m1
CCL3	C-C motif chemokine ligand 3	Hs00234142_m1
MSN	moesin	Hs00792607_mH
ACTG1	actin gamma 1	Hs03044422_g1
FCGR2A	Fc fragment of IgG receptor IIa	Hs00234969_m1
CXCL8	C-X-C motif chemokine ligand 8	Hs00174103_m1
CXCL1	C-X-C motif chemokine ligand 1	Hs00236937_m1
ALDOA	aldolase, fructose-bisphosphate A	Hs00605108_g1
ENO1	enolase 1	Hs00361415_m1
